# A Sequential Kalman-Newton-KM Framework for AIS and Radar Data Fusion in Restricted Inland Waterways

**DOI:** 10.3390/s26072255

**Published:** 2026-04-06

**Authors:** Huixia Shi, Dejun Wang, Longting Wei, Shan Liang

**Affiliations:** 1 School of Electronic Information Engineering, Chongqing Technology and Business Institute, Chongqing 401520, China; shx@cqtbi.edu.cn; 2 School of Automation, Chongqing University, Chongqing 400044, China; wdj@cqu.edu.cn; 3 Changjiang Wanzhou Waterway Division, Wanzhou 404020, China; 4 School of Transportation Engineering, East China Jiaotong University, Nanchang 330013, China; wlt@ecjtu.edu.cn

**Keywords:** AIS-radar data fusion, Kuhn-Munkres algorithm, restricted waterways, vessel tracking, sensor fusion

## Abstract

This paper presents a novel data fusion framework that integrates Automatic Identification System (AIS) data with radar surveillance for real-time vessel monitoring in inland restricted waterways. The approach exploits the complementarity between heterogeneous sensors: AIS provides semantic information with temporal sparsity, while radar offers high-frequency observations without vessel identity. The proposed solution combines Kalman filtering and Newton interpolation (K-N) for high-resolution AIS resampling, followed by optimal data association using the Kuhn-Munkres (KM) algorithm. By formulating data association as a global optimization problem, the framework achieves globally optimal sensor fusion while effectively handling data imbalance through virtual point augmentation. Experimental validation using real-world data demonstrates a matching accuracy of 94.2% in low-density scenarios and 80.1% in high-traffic conditions, with computational efficiency suitable for real-time deployment. The system performs consistently across different waterway geometries, although performance varies slightly between curved and straight channels. By fusing the high temporal resolution of radar data with the rich identity information from AIS, this framework enables more accurate and reliable vessel tracking, providing waterway authorities with enhanced situational awareness for improved traffic management and scheduling in restricted waterways.

## 1. Introduction

The shipping industry has emerged as a critical foundation for national economies, international trade, and social development as maritime power strategies continue to advance [1]. Due to its extensive coverage, substantial cargo capacity, and cost-effectiveness, maritime transportation maintains an irreplaceable position in global logistics [2]. According to China’s Ministry of Transport, waterway cargo volume reached 8.24 billion tons in 2021, representing an 8.2% year-on-year increase [3]. This rapid expansion has led to increased vessel traffic density, particularly in inland waterways [4], making traffic management and safety supervision increasingly critical [5,6].

To ensure navigation safety, regulations mandate that motorized vessels be equipped with the Automatic Identification System (AIS) [7]; while AIS provides rich semantic information (e.g., MMSI, speed, course), it suffers from temporal sparsity, low update frequencies, and potential data loss due to signal blockage or manual switch-off [8]. Conversely, Vessel Traffic Service (VTS) radar systems offer continuous, high-frequency surveillance but lack vessel identity information and are susceptible to clutter [9]. In complex inland environments, relying on a single sensor is insufficient. For instance, field investigations at the Yangtze River Maritime Safety Administration reveal that operators face significant challenges in: (1) associating radar tracks with AIS identities in crowded channels; (2) maintaining continuity when AIS signals are temporarily lost under bridges; and (3) predicting trajectories for collision avoidance. Therefore, the effective fusion of AIS and radar data is essential for constructing a reliable “digital twin” of waterway traffic [10,11,12].

The fusion of AIS and radar data has attracted considerable research attention [13], with methodologies generally categorized into probabilistic methods, clustering approaches, and learning-based techniques [14,15]. In the domain of probabilistic and statistical methods, Kazimierski and Stateczny analyzed decentralized fusion through covariance matrix calculations within ECDIS environments [16]. Gaglione et al. established a Bayesian framework utilizing belief propagation, demonstrating effectiveness in tracking multiple vessels [17]. Wang et al. proposed a weighted trajectory association algorithm based on motion state error independence [18]. Regarding clustering and fuzzy logic, Lin et al. implemented fuzzy C-mean clustering, leveraging trajectory characteristics for correlation under high ambiguity [19]. More recently, Artificial Intelligence (AI) approaches have gained prominence. Xiaorui developed a BP neural network for target association [20], while Rodger employed transfer learning on SAR images to classify vessel types for improved correlation [21]. Other studies have explored Long Short-Term Memory (LSTM) networks for trajectory modeling and Convolutional Neural Networks (CNN) for feature extraction [22,23].

Despite these contributions, critical gaps remain in applying these methods to inland restricted waterways [24]. First, temporal asynchrony remains a bottleneck; AIS updates (every 30 s to minutes) are significantly slower than radar sweeps (seconds), creating a mismatch that simple interpolation cannot accurately resolve in dynamic maneuvering scenarios [25]. Second, while deep learning models show promise, they often require massive annotated datasets and high computational resources ($O(n^{3})$ or higher), rendering them less suitable for real-time deployment on edge computing devices in VTS centers compared to lighter, deterministic algorithms [26]. Third, most existing algorithms assume a balanced number of targets, failing to robustly handle data imbalance (e.g., radar clutter creating false targets or AIS packet loss), which is common in narrow, curved inland channels [27,28].

To systematically address these limitations, this paper proposes a novel AIS-radar data fusion framework combining Kalman filtering, Newton interpolation, and the Kuhn-Munkres (KM) algorithm (K-N-KM). Unlike black-box AI models, this approach offers a mathematically explainable and computationally efficient solution suitable for real-time engineering applications. Table 1 presents a comparison of the proposed framework with existing methods.

The specific contributions of this paper are as follows:

1. High-Fidelity Temporal Synchronization: We propose a sequential Kalman-Newton (K-N) mechanism to resample sparse AIS data. This generates high-resolution trajectories that align precisely with radar timestamps, effectively bridging the temporal gap between sensors.
2. Global Optimization for Data Association: We formulate the matching process as a global assignment problem using the Kuhn-Munkres algorithm. This ensures globally optimal pairing rather than greedy local best-fits, significantly improving accuracy in high-density scenarios.
3. Robust Handling of Data Imbalance: We introduce a virtual point augmentation strategy within the KM framework. This allows the system to handle unequal numbers of AIS and radar targets (caused by sensor blind spots or clutter) without association failure.

The remainder of this paper is organized as follows: Section 2 details the mathematical formulation of the fusion problem. Section 3 presents the theoretical foundation of the K-N-KM framework. Section 4 describes the proposed methodology and implementation. Section 5 discusses experimental validation using real-world waterway data, and Section 6 concludes the paper.

## 2. Problem Description

Current maritime vessel monitoring relies on two primary sensor systems: AIS and radar. Each system offers distinct advantages but suffers from significant limitations when used independently. AIS provides comprehensive vessel information including identity, speed, heading, and destination, but suffers from inherent delay characteristics—vessel information updates occur only every 30 s to several minutes in inland waterways. This creates substantial discrepancies between vessels’ actual states and AIS-reported information, particularly in dynamic navigational environments such as restricted waterways, busy port areas, and narrow channels where real-time vessel positioning is critical for collision avoidance and traffic management.

Radar systems complement AIS by providing near-real-time positional data, with update frequencies as high as several times per second. This dramatically improves temporal resolution for vessel monitoring. However, radar detection faces challenges including environmental interference, clutter from river waves, and echo intensity variations, resulting in information errors and detection inconsistencies. More importantly, radar data lacks critical vessel identity information and detailed dynamic parameters, limiting its standalone effectiveness for comprehensive vessel tracking.

The fundamental challenge lies in effectively combining these complementary data sources to overcome their respective limitations. By fusing AIS and radar data, maritime authorities can simultaneously access vessel identity information while maintaining high-frequency positional updates, creating a more complete and accurate real-time operational picture of maritime traffic.

Assuming *m* AIS data points and *n* radar data points exist in the monitored area at a given time, the Euclidean distance between the *i*th AIS data point and the *j*th radar data point is defined as $c_{ij}⩾0$. The objective function for optimal matching becomes $min\left(Z\right)=\sum_{i}\sum_{j}c_{ij}x_{ij}$, where the decision variable is:

(1) $$x_{ij}=\left\{\begin{array}{cc} 1, & \mathrm{if}\;\mathrm{the}\;i\mathrm{th}\;\mathrm{AIS}\;\mathrm{data}\;\mathrm{point}\;\mathrm{corresponds}\;\mathrm{to}\;\mathrm{the}\;j\mathrm{th}\;\mathrm{radar}\;\mathrm{data}\;\mathrm{point}\\ 0, & \mathrm{otherwise} \end{array}\right.,$$

The matching process must satisfy the following constraints:

(2) $$\left\{\begin{array}{c} \sum_{i}x_{ij}=1,j=1,2,\ldots ,n\\ \sum_{j}x_{ij}=1,i=1,2,\ldots ,m\\ x_{ij}=1\;or\;0 \end{array}\right.$$

These constraints ensure a one-to-one correspondence between AIS and radar data points, with each AIS data point matching exactly one radar data point and vice versa. The solution to this optimization problem enables the integration of identity and dynamic information from AIS with the high-frequency positional updates from radar, creating a comprehensive and temporally accurate vessel tracking system.

## 3. Theoretical Foundation

This section establishes the mathematical and algorithmic foundation underlying the proposed K-N-KM fusion framework, which forms the core building blocks that enable effective integration of AIS and radar data in maritime surveillance applications. The theoretical framework is presented through three complementary components: Section 3.1 introduces the Kalman filtering algorithm for vessel state estimation and trajectory prediction under uncertainty; Section 3.2 presents the interpolation methodology for temporal synchronization between AIS predictions and radar observations; and Section 3.3 details the Kuhn-Munkres algorithm for optimal bipartite matching in multi-sensor data association. These three algorithmic approaches work synergistically to address the fundamental challenges of temporal asynchrony, trajectory prediction accuracy, and optimal sensor data fusion in maritime surveillance systems.

### 3.1. Kalman Filter

The Kalman filter is an optimal recursive estimator for linear systems with Gaussian noise. It operates through prediction and update cycles to combine predictions from system models with measurements from sensors.

For a discrete system with state equation $x_{k}=\phi_{k,k-1}x_{k-1}+\Gamma_{k-1}\omega_{k-1}$ and observation equation $Z_{k}=H_{k}x_{k}+\upsilon_{k}$, where $\omega_{k-1}$ and $\upsilon_{k}$ represent process and measurement noise with covariances $Q_{k}$ and $R_{k}$, respectively, the Kalman filter recursively calculates:

(3) $${\hat{x}}_{k/k-1}=\phi_{k,k-1}x_{k-1}$$

(4) $${\hat{P}}_{k/k-1}=\phi_{k,k-1}P_{k-1}\phi_{k,k-1}^{T}+\Gamma_{k-1}Q_{k-1}\Gamma_{k-1}^{T}$$

(5) $$K_{k}={\hat{P}}_{k/k-1}H_{k}^{T}{\left[H_{k}{\hat{P}}_{k/k-1}H_{k}^{T}+R_{k}\right]}^{-1}$$

(6) $$x_{k}={\hat{x}}_{k/k-1}+K_{k}\left[Z_{k}-H_{k}{\hat{x}}_{k/k-1}\right]$$

(7) $$P_{k}=\left[I-K_{k}H_{k}\right]\cdot {\hat{P}}_{k/k-1}$$

These equations represent state prediction, error covariance prediction, Kalman gain computation, state estimate update, and error covariance update, respectively. The standard Kalman filter’s linear dynamics assumption is appropriate for inland waterway vessels that typically operate at low speeds (5–15 knots) with short prediction intervals (30 s), where motion can be well-approximated as linear even during turns.

### 3.2. Interpolation Methods

Temporal synchronization between AIS and radar observations requires interpolation techniques to estimate vessel states at arbitrary time points. AIS updates arrive at irregular intervals, while radar scans operate at fixed cycles. To align these asynchronous data streams for fusion, interpolation methods are employed to predict AIS vessel positions at radar observation timestamps.

Linear interpolation approximates function values between known data points using first-order polynomials. For two points $\left(x_{0},y_{0}\right)$ and $\left(x_{1},y_{1}\right)$, the interpolating function is:

(8) $$\varphi_{1}\left(x\right)=y_{0}+\frac{y_{1}-y_{0}}{x_{1}-x_{0}}\left(x-x_{0}\right)$$

This method provides computational efficiency for short-term predictions where vessel motion can be reasonably approximated as linear.

Newton interpolation offers computational inheritance when adding new interpolation points, making it suitable for adaptive prediction windows. Using divided differences, the Newton interpolation formula is:

(9) $$\begin{array}{cc} N_{n}\left(x\right) & =f\left[x_{0}\right]+f\left[x_{0},x_{1}\right]\left(x-x_{0}\right)\\ \; & +f\left[x_{0},x_{1},x_{2}\right]\left(x-x_{0}\right)\left(x-x_{1}\right)+\cdots \end{array}$$

where $f[x_{0},x_{1},\ldots ,x_{k}]$ represents the *k*-th order divided difference. This higher-order approximation captures non-linear vessel trajectories more accurately during maneuvering scenarios.

### 3.3. Kuhn-Munkres Algorithm

The Kuhn-Munkres (KM) algorithm, also known as the Hungarian algorithm, finds optimal matchings in weighted bipartite graphs. For the data fusion problem with AIS and radar data points, a bipartite graph is formulated where the edge weight between AIS point *i* and radar point *j* is represented by their distance $c_{ij}$.

The algorithm maintains feasible vertex labelings Lx(x) and Ly(y) satisfying Lx(x)+Ly(y)≥weight(x,y) for all edges, and constructs equality subgraphs containing only edges where Lx(x)+Ly(y)=weight(x,y). It iteratively modifies these labelings to increase viable edges until finding a perfect matching with maximum weight.

For matching problem with objective function $\min Z=\sum_{i}\sum_{j}c_{ij}x_{ij}$ and constraints ensuring one-to-one correspondence between AIS and radar data points, the KM algorithm provides an optimal solution by identifying the minimum-cost assignment.

## 4. Proposed K-N-KM Fusion Framework

This section presents the detailed methodology of the proposed K-N-KM fusion framework for integrating AIS and radar data in inland waterway navigation. The framework addresses the fundamental challenge of temporal asynchrony between heterogeneous sensor data through a three-stage algorithmic approach that enables accurate and efficient data association. The section is organized as follows: Section 4.1 provides an overview of the complete framework architecture, demonstrating how the Kalman filtering, Newton interpolation, and modified Kuhn-Munkres components work synergistically to achieve robust sensor fusion; Section 4.2 details the AIS trajectory prediction methodology using Kalman filtering for vessel state estimation under uncertainty; Section 4.3 describes the temporal resolution enhancement process through Newton interpolation, explaining how high-frequency AIS positions are generated to match radar sampling rates; Section 4.4 presents the modified Kuhn-Munkres algorithm for optimal bipartite matching in multi-sensor data association.

### 4.1. Framework Architecture

**Figure 1:**
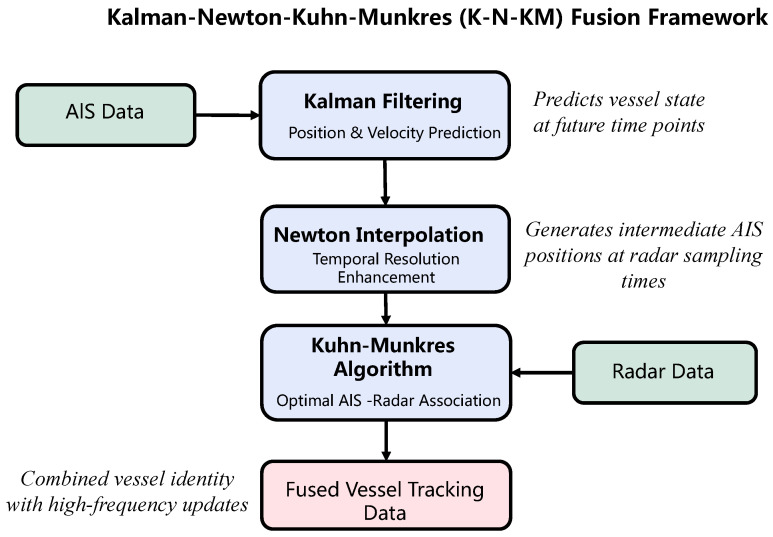
Overview of the proposed K-N-KM fusion framework.

The proposed Kalman-Newton-Kuhn-Munkres (K-N-KM) fusion framework addresses the fundamental challenge of integrating low-frequency AIS data with high-frequency radar data for vessel tracking. As illustrated in Figure 1, the framework consists of three key components: (1) AIS trajectory prediction using Kalman filtering, (2) temporal resolution enhancement through Newton interpolation, and (3) optimal AIS-radar data association via the Kuhn-Munkres algorithm.

Unlike previous approaches that directly match raw AIS and radar data or rely on computationally intensive neural networks, this method leverages the complementary strengths of classical algorithms in a novel integrated framework. The primary innovation lies in the sequential application of these algorithms to address the specific challenges of maritime data fusion in inland waterways, where vessel density and waterway geometry create unique tracking challenges. While this work focuses on AIS-radar fusion as the standard configuration in maritime surveillance, the framework’s mathematical foundation provides extensibility to additional sensors where operationally viable.

### 4.2. Kalman Filtering for Trajectory Prediction

The first component of the framework employs Kalman filtering to predict vessel trajectories from sparse AIS updates. Each vessel’s motion is modeled using a state vector comprising position and velocity components:

(10) $$G\left(t\right)={\left[x,y,dx,dy\right]}^{T}$$

where (x,y) represents the vessel’s position coordinates and (dx,dy) represents its velocity components. The Kalman filter’s error covariance matrix $P_{k}$ inherently quantifies prediction uncertainties, providing confidence estimates for both position and velocity components at each time step.

For inland waterway vessels, the state transition matrix is defined to account for the constrained maneuverability typical in narrow channels:

(11) $$A=\left[\begin{array}{cccc} 1 & 0 & 1 & 0\\ 0 & 1 & 0 & 1\\ 0 & 0 & 1 & 0\\ 0 & 0 & 0 & 1 \end{array}\right]$$

Furthermore, the measurement matrix that relates the state vector to observable quantities:

(12) $$H=\left[\begin{array}{cccc} 1 & 0 & 0 & 0\\ 0 & 1 & 0 & 0 \end{array}\right]$$

Through empirical analysis of vessel movement patterns in restricted waterways, optimal process and measurement noise covariance matrices were determined:

(13) $$Q=0.003\cdot \left[\begin{array}{cccc} 1 & 0 & 0 & 0\\ 0 & 1 & 0 & 0\\ 0 & 0 & 1 & 0\\ 0 & 0 & 0 & 1 \end{array}\right]$$

(14) $$R=0.5\cdot \left[\begin{array}{cc} 1 & 0\\ 0 & 1 \end{array}\right]$$

The selection of these covariance parameters was guided by the physical characteristics of inland waterway navigation. The small process noise (Q = 0.003) reflects the relatively predictable motion of inland vessels at low speeds (5–15 knots), while the measurement noise (R = 0.5) corresponds to typical GPS positioning errors of 10–15 m in commercial AIS. These values were validated through sensitivity analysis on a separate calibration subset, which confirmed stable matching performance for Q values between 0.002 and 0.005 and R values between 0.3 and 0.6. Fixed parameters were adopted to ensure deterministic, reproducible behavior suitable for safety-critical maritime applications.

Given these parameters, this method predicts vessel positions at future time points through the recursive Kalman filtering process described in Section 3.1. The key innovation is the optimization of these parameters specifically for inland waterway vessel movement patterns, which differ significantly from open-sea navigation. The process noise covariance *Q* and measurement noise covariance *R* matrices were empirically tuned to reflect uncertainty characteristics of vessel movements in restricted waterways, with the resulting covariance matrix $P_{k}$ providing quantitative measures of estimation reliability.

### 4.3. Newton Interpolation for Temporal Alignment

Even though Kalman filtering provides accurate predictions at future AIS update times, radar systems operate at much higher frequencies. To align these different temporal resolutions, Newton interpolation is applied between consecutive Kalman-predicted positions.

For each vessel, separate interpolation functions are constructed for latitude and longitude coordinates. Given the predicted position at time *t* and t+Δt, intermediate positions are generated at radar sampling times $t_{i}$ where $t<t_{i}<t+\Delta t$.

The method computes the divided differences for latitude coordinates:

(15) $$Lat\left[t_{0},t_{1}\right]=\frac{Lat\left(t_{1}\right)-Lat\left(t_{0}\right)}{t_{1}-t_{0}}$$

Higher-order differences are recursively derived:

(16) $$Lat\left[t_{0},\ldots ,t_{k+1}\right]=\frac{Lat\left[t_{1},\ldots ,t_{k+1}\right]-Lat\left[t_{0},\ldots ,t_{k}\right]}{t_{k+1}-t_{0}}$$

The resulting Newton interpolation polynomial provides position estimates at any time within the interval:

(17) $$\begin{array}{c} N_{n}^{lat}\left(t\right)=Lat\left[t_{0}\right]+Lat\left[t_{0},t_{1}\right]\left(t-t_{0}\right)+\cdots +Lat\left[t_{0},\ldots ,t_{n}\right]\prod_{i=0}^{n-1}\left(t-t_{i}\right) \end{array}$$

In the proposed framework, Kalman filtering and Newton interpolation serve complementary roles: the Kalman filter predicts vessel states at discrete future AIS update times, while Newton interpolation generates intermediate positions between these predictions to match radar sampling rates. Newton interpolation was selected over alternatives due to its computational inheritance—unlike cubic spline interpolation requiring full recalculation when new data arrives, only additional terms need calculation for new AIS updates. Compared to Lagrange interpolation, Newton’s divided difference formulation maintains numerical stability with non-uniformly spaced time samples common in AIS data. Since inland waterway vessels operate at low speeds (typically less than 15 knots) with short interpolation intervals, vessel motion between predicted points remains approximately linear, ensuring physically plausible trajectories. In this implementation, quadratic interpolation (*n* = 2) was adopted, providing sufficient flexibility to capture trajectory curvature while avoiding numerical artifacts associated with higher-order polynomials.

### 4.4. Modified KM Algorithm for Optimal Matching

The final component of the framework addresses the data association problem: determining which radar detection corresponds to which AIS-identified vessel. This is formulated as a minimum-cost bipartite matching problem and solved using a modified Kuhn-Munkres algorithm.

Given the sets of interpolated AIS positions $A=\{A_{1}\left(x_{1},y_{1}\right),A_{2}\left(x_{2},y_{2}\right),\ldots ,A_{m}\left(x_{m},y_{m}\right)\}$ and radar detections $R=\{R_{1}\left(x_{1},y_{1}\right),R_{2}\left(x_{2},y_{2}\right),\ldots ,R_{n}\left(x_{n},y_{n}\right)\}$ at a specific time point, the method constructs a distance matrix $M_{m\times n}$ where each element represents the Euclidean distance between an AIS position and a radar detection. This distance-based formulation implicitly encodes association confidence, where smaller distances indicate higher confidence in potential vessel pairings, and the optimal matching solution represents the highest-confidence assignment configuration given the available data.

Since the KM algorithm maximizes total weight in assignments while the objective is to minimize distances, the distance matrix is transformed by: (1) finding the maximum distance value $H=\max\{M_{i,j}\}$; (2) creating a maximum matrix of the same dimensions with all elements equal to *H*; and (3) subtracting the original distance matrix from this maximum matrix to obtain $Y_{m\times n}$.

A key innovation in this approach is the handling of unequal numbers of AIS and radar data points. When m>n (more AIS points than radar detections), the algorithm supplements with virtual radar points and assigns zero weights to connections with these virtual points. Virtual radar points are assigned zero cost and serve as algorithmic placeholders without introducing bias, as they do not affect the optimization of actual radar-AIS associations. This creates a square computation matrix $T_{m\times m}$ suitable for the KM algorithm while ensuring that unmatched AIS points are correctly identified. It should be noted that the virtual point augmentation does not require a predefined distance threshold. When m > n, virtual radar points with zero-cost edges are added to create a balanced bipartite graph. The KM algorithm’s global optimization naturally assigns AIS points without corresponding radar detections to these virtual points, effectively identifying unmatched targets without arbitrary threshold selection.

Algorithm 1 outlines the modified KM implementation, which incorporates specialized conflict resolution procedures for maritime data association contexts.
**Algorithm 1** Modified Kuhn-Munkres Algorithm for AIS-Radar Association**Require:** AIS positions $A=\{A_{1},A_{2},\ldots ,A_{m}\}$, radar detections $R=\{R_{1},R_{2},\ldots ,R_{n}\}$, transformed distance matrix $T_{m\times m}$, adjustment parameter d=0.1**Ensure:** Optimal matching A←R  1:**for** i=1,2,…,m **do**  2:      $A_{i}^{*}=\max\left\{T_{i,1},T_{i,2},\ldots ,T_{i,m}\right\}$, $R_{i}^{*}=0$  3:$z_{i,j}=A_{i}^{*}+R_{j}^{*}-T_{i,j}$  4:**while** not every AIS point has a radar match **do**  5:      **for** i=1,2,…,m **do**  6:            **for** j=1,2,…,m **do**  7:                  **if** $z_{i,j}=0$ **then**  8:                        match $A_{i}\leftarrow R_{j}$  9:                        **break**10:            **if** matching contradiction occurs **then**11:                  Identify contradicting AIS points $\alpha_{1},\alpha_{2}$ and radar point $\beta_{1}$12:                  **if** $\exists p\in \{row\;\alpha_{2}\}$ and $z_{\alpha_{2},p}=0$ **then**13:                        $A_{\alpha_{2}}\leftarrow R_{p}$, $A_{\alpha_{1}}\leftarrow R_{\beta_{1}}$14:                  **else if** $\exists q\in \{row\;\alpha_{1}\}$ and $z_{\alpha_{1},q}=0$ **then**15:                        $A_{\alpha_{1}}\leftarrow R_{q}$, $A_{\alpha_{2}}\leftarrow R_{\beta_{1}}$16:                  **else**17:                        $\delta =\min\{z_{i,j}|i\in S,j\notin T\}$18:                        $A_{i}^{*}=A_{i}^{*}-\delta$ for i∈S19:                        $R_{j}^{*}=R_{j}^{*}+\delta$ for j∈T20:                        Update slack values $z_{i,j}$21:**return** all matches A←R

## 5. Experimental Setup and Results Analysis

This section presents an experimental evaluation of the proposed K-N-KM fusion framework using real-world data. The evaluation demonstrates the framework’s effectiveness in addressing temporal asynchrony challenges and achieving accurate AIS-radar data association under various operational conditions.

### 5.1. Experimental Setup

Experiments were conducted using real-world data from the Shenbeizui Restricted Waterway in Sichuan Province, China, collected between 6 September and 23 October 2024. The raw dataset contains 7,596,274 AIS records and 2,593,032 radar detections from 468 vessels. The AIS data was received with an average update interval of approximately 30 s, while the radar system operated with a scan period of 1 s. After spatial and quality filtering to remove out-of-zone transmissions and noisy detections, 30,000 high-quality time points (10,000 per traffic density category) were randomly sampled for balanced evaluation. All experiments were executed on a computer with Intel i7-11700K CPU and 16 GB RAM. Traffic density is categorized into three levels based on instantaneous vessel counts in the monitoring area: low density (<10 vessels), medium density (10–30 vessels), and high density (>30 vessels).

### 5.2. Data Preprocessing

**Figure 2:**
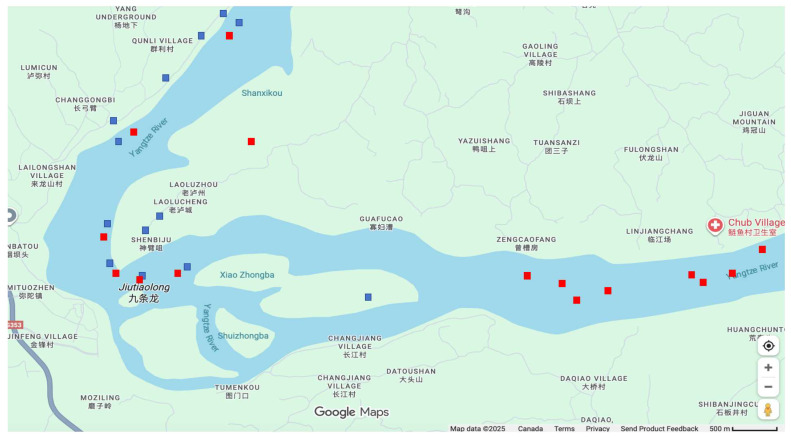
Original data distribution showing AIS points (red) and radar detections (blue).

Maritime sensor data contains anomalies due to environmental interference and signal transmission failures, which must be filtered before fusion. Figure 2 shows the raw data distribution. To ensure data quality for the proposed K-N-KM fusion framework, a systematic preprocessing pipeline was implemented with three steps.

Standard data cleaning procedures were applied to both AIS and radar streams. Specifically, following criteria were applied: (1) duplicate records with identical MMSI, timestamp, and position were removed; (2) AIS records with invalid MMSIs were filtered; (3) position jumps exceeding 500 m between consecutive updates within a 30 s interval were removed; (4) speed values exceeding 30 knots, which are unrealistic for inland vessels, were excluded; and (5) stationary objects identified by speed below 0.5 knots maintained for more than 10 consecutive minutes were eliminated to focus on active navigating vessels. All timestamps were converted to UTC for temporal alignment, with radar timestamps serving as the common time reference for subsequent fusion processing.

### 5.3. Performance Evaluation

This subsection presents comprehensive performance analysis of the K-N-KM framework across multiple evaluation dimensions: trajectory enhancement quality, matching accuracy under varying conditions, computational efficiency, and environmental factor impacts.

#### 5.3.1. AIS Trajectory Enhancement Results

**Figure 3:**
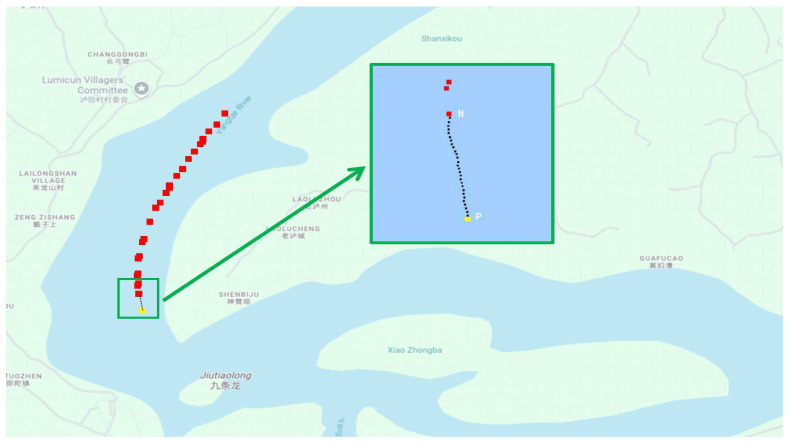
AIS trajectory enhancement showing actual positions (red), Kalman predictions (yellow), and interpolated positions (black). The inset provides a detailed view of interpolated trajectory points between AIS updates.

The K-N resampling method successfully generated high-resolution AIS trajectories that align with the temporal frequency of radar detections. Figure 3 illustrates the trajectory enhancement results, where red points represent actual AIS positions transmitted by vessels, yellow points indicate Kalman-predicted positions at future time t+Δt, and black points show Newton-interpolated positions at intermediate time steps. The inset provides a magnified view of a selected region, demonstrating how the method effectively maintains trajectory continuity while increasing temporal resolution to match radar sampling rates.

The enhanced temporal resolution achieved through K-N method enables more accurate matching with radar data compared to using raw AIS updates. For any time point within the prediction interval, the method generates corresponding AIS position estimates that can be directly compared with concurrent radar detections.

#### 5.3.2. Matching Accuracy Analysis

To establish performance benchmarks, the K-N-KM framework was compared against traditional Nearest Neighbor and Particle Filter approaches across varying vessel densities. All methods were evaluated on the identical dataset using the same evaluation metrics under consistent experimental conditions. Table 2 presents matching accuracy comparison results.

The K-N-KM framework consistently outperforms both baseline methods across all density categories, achieving improvements of 8–22 percentage points. Notably, the framework maintains 80.1% accuracy even in challenging high-density scenarios exceeding 30 vessels, where traditional nearest neighbor matching degrades to 58.7% due to ambiguous spatial proximity and particle filter approaches reach only 76.2%, limited by particle degeneracy in crowded environments.

To provide comprehensive performance evaluation, detailed analysis was conducted using precision, recall, and F1 score metrics. For each test scenario, confusion matrices were constructed recording true positive (TP), false positive (FP), true negative (TN), and false negative (FN) events. Based on these values, precision (P = TP/(TP + FP)), recall (R = TP/(TP + FN)), and F1 score (F1 = 2PR/(P + R)) were calculated.

Table 3 presents granular performance metrics across five detailed vessel count ranges, revealing a consistent degradation pattern as traffic density increases, i.e., F1 scores decreasing gradually from 97.0% in sparse traffic to 81.9% in dense scenarios (50–100 vessels).

**Figure 4:**
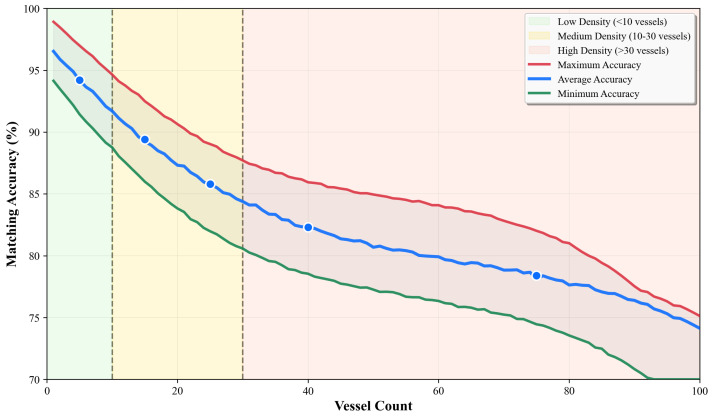
Matching accuracy versus vessel density, showing maximum (red), average (blue), and minimum (green) performance across different traffic density levels indicated by background shading.

Figure 4 presents the accuracy results as a function of vessel density across the full operational range (1–100 vessels), demonstrating a consistent degradation pattern as waterway traffic increases. The three curves represent maximum, average, and minimum accuracy observed across different spatial distribution patterns. The color-coded background regions indicate density categories: low density (<10 vessels, green), medium density (10–30 vessels, yellow), and high density (>30 vessels, orange).

The performance variation between best and worst cases (red and green lines) illustrates the significant impact of vessel distribution patterns on matching performance. In optimal scenarios (red line), the framework achieves 97% accuracy in low-density conditions and maintains above 82% even in high-density situations when vessels maintain scattered and uniform spatial distribution. Conversely, worst-case scenarios (green line) occur when vessels navigate in tightly clustered formations, resulting in accuracy degradation to approximately 91% for low-density and 75% for high-density scenarios. The average performance (blue line) demonstrates robust accuracy of 94.2% for sparse traffic, degrading gracefully to 80.1% as density exceeds 30 vessels, with marked data points representing empirically measured values from Table 3. The performance degradation in high-density scenarios can be attributed to three typical failures: (1) spatial ambiguity when multiple vessels navigate within close distances, leading to association conflicts; (2) radar occlusion caused by larger vessels blocking the detection of smaller ones; and (3) temporal misalignment due to AIS transmission delays.

#### 5.3.3. Computational Efficiency

To validate the framework’s suitability for real-time deployment, performance benchmarking was conducted. Each scenario was evaluated over 100 iterations to ensure statistical reliability. Table 4 presents the execution times for complete processing cycles across different vessel counts.

The results reveal a distinct non-linear growth in total processing time as the number of vessels increases. As shown in Table 4, the KM algorithm’s computational cost rises sharply, accounting for over 90% of the total time in high-density scenarios (100 vessels).

Despite the algorithmic complexity, the framework demonstrates robust real-time performance. In Shenbeizui channel, the simultaneous presence of 100 vessels is an extreme stress test scenario that far exceeds typical traffic density. Even under this maximum load, the total execution time remains approximately 416 ms. Regarding memory consumption, the primary overhead arises from storing the distance matrix (O(mn)) and the KM algorithm’s auxiliary structures. Since the algorithm processes each time point independently without maintaining historical trajectories, memory usage remains modest and scales only with vessel count. These efficiency characteristics ensure suitability for deployment on standard edge devices in VTS centers.

#### 5.3.4. Impact of Environmental Factors

**Figure 5:**
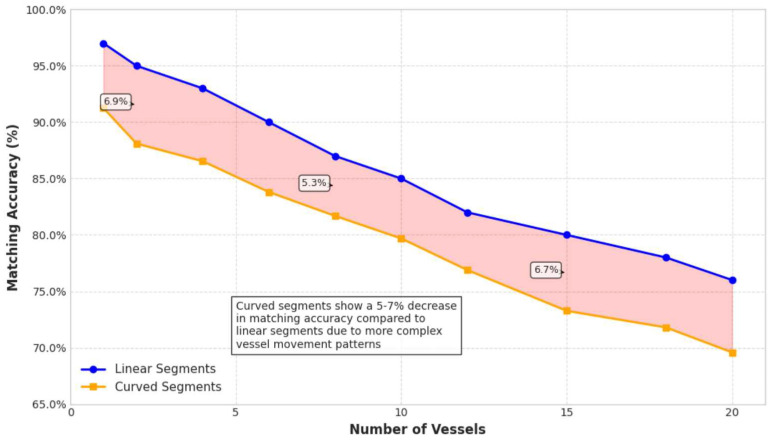
Matching accuracy comparison between linear (blue) and curved (orange) waterway segments.

The system’s performance was evaluated across different waterway configurations, with sections categorized as either linear or curved. Figure 5 compares matching accuracy between these two configurations based on analysis of 10,000 time points for each type (20,000 total evaluation instances).

To validate the statistical significance of observed differences, a two-sample *t*-test comparing matching accuracy between linear segments (*n* = 847 test cases, mean accuracy = 89.3%, σ = 4.2%) and curved segments (*n* = 653 test cases, mean accuracy = 85.7%, σ=5.8%) was conducted. The results confirm a statistically significant difference (*t* = 11.47, p<0.001, 95% CI: 2.8–4.4%), indicating that waterway geometry meaningfully impacts fusion performance.

To explore additional geometric factors, statistical variance analysis was applied to examine waterway width, curvature radius, and their interaction with vessel density. As shown in Table 5, curvature radius significantly affects performance (F(2650) = 23.8, p<0.001), with tighter curves showing 6.2% lower accuracy than gradual curves. Waterway width also demonstrates significant impact (F(2,1497) = 15.4, p<0.001), with narrow waterways experiencing 3.7% reduced accuracy due to increased radar multipath effects from nearby banks.

Correlation analysis reveals that curvature radius (r = −0.34, p<0.001) and waterway width (r = 0.28, p<0.001) are significant predictors of fusion accuracy, with interaction effects between geometry and vessel density showing that curved narrow waterways exhibit the most pronounced performance degradation in high-traffic scenarios. This difference stems from the distinct navigational characteristics of curved waterways: (1) faster currents and narrower passages in curved segments, (2) reduced vessel speeds and smaller inter-vessel distances, and (3) more complex traffic patterns requiring coordinated navigation. In linear segments, vessels typically maintain more uniform speeds and greater separation distances, creating more favorable conditions for the distance-based matching algorithm.

### 5.4. Detailed Case Study

To demonstrate the practical operation of the proposed K-N-KM framework, this subsection presents a detailed walkthrough of the complete matching process using a representative time slice from the dataset. The selected period spans from 1:10:47 to 1:10:49, during which six vessels with active AIS transmissions were navigating through the monitored waterway section. This case study illustrates how the framework systematically processes raw sensor data to achieve accurate vessel identification and tracking.

Table 6 presents the raw input data for this scenario, displaying radar detections (left columns) and AIS transmissions (right columns) captured at each time point. An important observation from this table is the temporal mismatch characteristic of real-world maritime surveillance: while radar consistently detects five targets at 1:10:48 and 1:10:49, AIS data is available for six vessels throughout the period, with vessel 413931559 lacking corresponding radar detection at 1:10:47. This exemplifies the data imbalance challenge that the modified KM algorithm is designed to address.

Following the framework methodology outlined in Section 4, the matching process begins with distance matrix construction. For each AIS-radar pair, the Euclidean distance is calculated using their geographic coordinates. Table 7 presents the resulting distance matrix $M_{6\times 5}$, where rows correspond to the six AIS-equipped vessels (identified by MMSI) and columns represent the five radar detections. The matrix values, expressed in meters, reveal distinct proximity patterns: vessel 413762351 exhibits minimal distance (91.0 m) to radar 1, while vessel 413811419 is closest to radar 3 (18.4 m). These spatial relationships form the basis for the subsequent optimization problem.

Since the KM algorithm maximizes edge weights while the objective is to minimize distances, a transformation step is required. As detailed in Section 4.4, this is accomplished by subtracting each distance value from the maximum distance in the matrix (4718.3 m for this case). Additionally, to handle the data imbalance where six AIS vessels correspond to only five radar detections, a virtual radar column is appended with zero-cost assignments. Table 8 shows the transformed computation matrix $T_{6\times 6}$ ready for KM algorithm processing. In this transformed matrix, larger values now indicate more favorable associations, and the virtual radar column enables the algorithm to identify which vessel lacks a corresponding radar detection.

Applying the modified KM algorithm (Algorithm 1) to the computation matrix yields the optimal one-to-one assignment. Table 9 presents the matching results in binary matrix form, where each cell value of 1 indicates an established correspondence between the AIS vessel (row) and radar detection (column). The algorithm successfully identifies five real vessel-radar associations while assigning vessel 413931559 to the virtual radar point, indicating this vessel was not detected by radar during this time slice due to radar occlusion or the vessel being outside the effective detection range.

**Figure 6:**
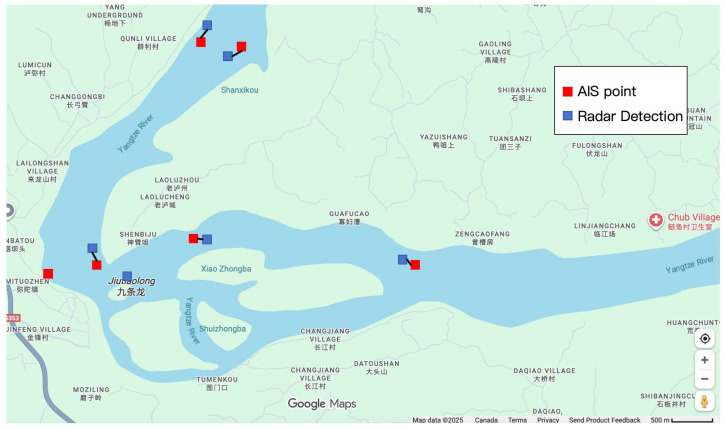
Spatial visualization of AIS-radar matching results. Blue points represent radar detections, red points represent AIS positions. The close proximity of matched pairs validates the algorithmic assignments from Table 9.

To validate these algorithmic matching results against ground truth, Figure 6 provides spatial visualization of the matched AIS-radar pairs. Blue points represent radar detections while red points denote AIS positions, with the close spatial proximity between color-matched pairs confirming the accuracy of the KM algorithm’s assignments. Notably, the five successfully matched vessels exhibit position discrepancies of less than 100 m between their AIS and radar coordinates, well within the expected accuracy range given GPS and radar measurement uncertainties. This case study demonstrates that the framework correctly handles real-world challenges including data imbalance and varying vessel densities while maintaining matching accuracy consistent with the overall system performance reported in Section 5.3.

## 6. Conclusions

This paper presented a novel K-N-KM framework for integrating AIS and radar data in restricted inland waterways through the synergistic combination of Kalman filtering, Newton interpolation, and the Kuhn-Munkres algorithm. The framework effectively addresses the fundamental challenge of temporal asynchrony between heterogeneous sensor systems while preserving critical vessel identity information and achieving enhanced temporal resolution for real-time maritime surveillance applications. Experimental validation using real-world data from the Shenbeizui Restricted Waterway demonstrated robust performance across varying operational conditions. The system achieved a matching accuracy of 94.2% in low-density scenarios (<10 vessels) and maintained 80.1% accuracy in high-density conditions (>30 vessels). Performance analysis across 30,000 evaluation instances confirmed the framework’s reliability, with computational efficiency suitable for immediate deployment in existing vessel traffic service centers.

Several limitations exist in this study. The framework experiences performance degradation in high-density scenarios due to spatial proximity conflicts and radar occlusion events, with matching accuracy decreasing to approximately 80%. Additionally, curved waterway segments show reduced performance (85.7% accuracy) compared to linear Sections (89.3% accuracy), indicating geometric sensitivity that may limit effectiveness in highly meandering waterways. Furthermore, current implementation does not model environmental conditions such as weather or tidal effects, relying instead on the Kalman filter’s process noise model to implicitly account for variations. The framework processes AIS and radar inputs through standard interfaces and maintains computational efficiency compatible with typical VTS edge devices. While validated on the Yangtze River, the framework is mathematically generic and not restricted to specific waterway types. Adaptation to other environments would primarily require recalibration of noise covariance parameters to account for different vessel dynamics. Future research directions include extending the proposed K-N-KM framework to additional sensing modalities (e.g., LiDAR, vision cameras), leveraging its modality-agnostic formulation to support multi-sensor fusion while maintaining the computational efficiency required for real-time deployment.

## Figures and Tables

**Table 1:** Comparison of Existing AIS-Radar Fusion Methods with Proposed K-N-KM Framework.

Method	Temporal Asynchrony Handling	Computational Complexity	Unequal Data Handling	Key Limitation
Fuzzy C-mean [19]	Moderate (Fuzzy logic)	High $O(n^{2}m^{2})$	Limited	Performance drops in dense traffic
Weighted Trajectory [18]	Limited (Direct matching)	Moderate $O(n^{2}m)$	No	Requires equal data points
Neural Networks (BP/CNN) [20]	Good (Learned patterns)	Very High $O(n^{3})$	Yes	High training cost; “Black box” nature
NN/Greedy Search [23]	Limited	Low O(nm)	No	Local optima; prone to mismatch errors
Proposed K-N-KM	Excellent (K-N Resampling)	Moderate $O(nm^{2})$	Yes (Virtual Points)	Slightly reduced performance in sharp curves

Note: In the complexity notation, *m* denotes the number of AIS data points and *n* denotes the number of radar data points in the monitored area.

**Table 2:** Performance Comparison with Baseline Methods.

Traffic Density	Nearest Neighbor	Particle Filter	K-N-KM
Low density (<10 vessels)	86.3%	91.7%	94.2%
Medium density (10–30 vessels)	72.4%	84.9%	87.6%
High density (>30 vessels)	58.7%	76.2%	80.1%

**Table 3:** Detailed Matching Quality Metrics under Different Vessel Counts.

Vessel Count	Accuracy	Precision	Recall	F1 Score
<10 vessels	94.2%	96.8%	97.2%	97.0%
10–20 vessels	89.4%	92.1%	93.5%	92.8%
20–30 vessels	85.8%	88.6%	89.8%	89.2%
30–50 vessels	82.3%	84.9%	86.1%	85.5%
50–100 vessels	78.4%	81.2%	82.6%	81.9%

**Table 4:** Execution Time for Different Vessel Counts (Mean ± SD).

Vessel Count	Total (ms)	KM Algorithm (ms)	Other Steps (ms)
10	4.8 ± 0.5	2.1 ± 0.3	2.7 ± 0.3
30	24.5 ± 2.1	16.8 ± 1.8	7.7 ± 0.6
50	76.2 ± 5.4	62.4 ± 4.9	13.8 ± 1.1
70	185.4 ± 12.6	164.5 ± 11.2	20.9 ± 1.8
100	415.8 ± 28.3	382.1 ± 26.5	33.7 ± 2.5

**Table 5:** Statistical Analysis of Geometric Factors on Fusion Performance.

Geometric Factor	Categories	Mean Accuracy (%)	F/*p*-Value
Waterway Width	Narrow (<150 m)	84.6 ± 5.9	F(2,1497) = 15.4, p<0.001
	Medium (150–300 m)	87.2 ± 4.8	
	Wide (>300m)	88.3 ± 4.2	
Curvature Radius	Tight (<500 m)	82.1 ± 6.4	F(2650) = 23.8, p<0.001
	Moderate (500–1000 m)	86.8 ± 5.1	
	Gradual (>1000 m)	88.3 ± 4.7	

**Table 6:** Radar Detections and AIS Transmissions at Selected Time Points.

Time	Radar Data			AIS Data		
	Longitude	Latitude	Radar ID	Longitude	Latitude	MMSI
1:10:47	105.644173	28.906542	radar 1	105.6451068	28.90659273	413762351
1:10:47	105.644516	28.899954	radar 2	105.6436365	28.89995964	413848724
1:10:47	105.629044	28.872293	radar 3	105.6292252	28.8723573	413811419
1:10:47	105.666328	28.873638	radar 4	105.6666647	28.87313666	413817788
1:10:47	-	-	-	105.6408968	28.87516618	413867146
1:10:47	-	-	-	105.6180445	28.87084834	413931559
1:10:48	105.644203	28.90658	radar 1	105.6451209	28.90661842	413762351
1:10:48	105.644524	28.899937	radar 2	105.6436359	28.89995993	413848724
1:10:48	105.629082	28.872322	radar 3	105.6292549	28.87237078	413811419
1:10:48	105.666313	28.873646	radar 4	105.6666476	28.87314192	413817788
1:10:48	105.640839	28.875477	radar 5	105.6408958	28.87517643	413867146
1:10:48	-	-	-	105.6180453	28.87084914	413931559
1:10:49	105.644203	28.906607	radar 1	105.6451349	28.90664411	413762351
1:10:49	105.644501	28.899944	radar 2	105.6436354	28.89996023	413848724
1:10:49	105.629105	28.872335	radar 3	105.6292847	28.87238426	413811419
1:10:49	105.666306	28.873646	radar 4	105.6666304	28.87314717	413817788
1:10:49	105.640839	28.875471	radar 5	105.6408949	28.87518668	413867146
1:10:49	-	-	-	105.6180461	28.87084995	413931559

Note: Dashes indicate no radar detection for the corresponding time point.

**Table 7:** Distance Matrix Between AIS and Radar Positions (meters).

MMSI/Radar ID	Radar 1	Radar 2	Radar 3	Radar 4	Radar 5
413762351	91.0	745.2	4111.5	4200.0	3480.5
413811419	4062.6	3396.1	18.4	3614.4	1177.2
413817788	4305.6	3671.6	3662.0	63.7	2530.0
413848724	738.8	84.4	3374.0	3660.1	2728.0
413931559	4713.4	4130.1	1091.4	4718.3	2281.1
413867146	3497.4	2766.4	1192.8	2484.9	32.1

**Table 8:** Transformed Computation Matrix for KM Algorithm.

MMSI/Radar ID	Radar 1	Radar 2	Radar 3	Radar 4	Radar 5	Virtual Radar
413762351	4627.3	3973.1	606.8	518.3	1237.8	0
413811419	655.7	1322.2	4699.9	1103.9	3541.1	0
413817788	412.7	1046.7	1056.3	4654.6	2188.3	0
413848724	3979.5	4633.9	1344.3	1058.2	1990.3	0
413931559	4.9	588.2	3626.9	0	2437.2	0
413867146	1220.9	1951.9	3525.5	2233.4	4686.2	0

**Table 9:** Optimal AIS-Radar Matching Results.

MMSI/Radar ID	Radar 1	Radar 2	Radar 3	Radar 4	Radar 5	Virtual Radar
413762351	1	0	0	0	0	0
413811419	0	0	1	0	0	0
413817788	0	0	0	1	0	0
413848724	0	1	0	0	0	0
413931559	0	0	0	0	0	1
413867146	0	0	0	0	1	0

## Data Availability

The data presented in this study are not publicly available due to restrictions imposed by the data provider (Changjiang Waterway Bureau).
